# The Cost-Benefit of Aging: Financial Capability and Well-Being across Age Groups in Brazil

**DOI:** 10.1155/2023/2020189

**Published:** 2023-10-10

**Authors:** Eduarda A. S. R. G. da Silva, César A. T. Silva

**Affiliations:** School of Economy, Business Administration, Accounting and Public Policies (FACE), University of Brasilia (UnB), Brasília, Brazil

## Abstract

A large proportion of older persons in developing countries do not have access to pension, which also constrains their ability to afford healthcare services and entails extensive challenges to the well-being of older people. This study aimed to analyze the financial preparedness of different age groups for retirement in Brazil. Data were derived from a survey to empirically validate the proposed relationships between *preparedness for retirement* and *resilience for the future* (financial well-being (FWB) outcomes) on the one hand and among *demographic and socioeconomic* aspects, *behaviors and attitudes*, *knowledge and experience*, and “key” *psychological factors* on the other hand. The sample consisted of 412 individuals aged between 22 and 79 years. FWB was measured using the financial capability and well-being model and regressed on a number of sociodemographic and psychological variables using linear regression analyses. The results demonstrated that *preparedness for retirement* was strongly related to older age. Additionally, age was correlated with *resilience for the future* close to zero, which indicates no relationship. *Knowledge* and the *psychological factors* of self-control and confidence were positively and strongly related to better *financial behavior* for all age groups. In addition, g*rit* and *resilience for the future* were positively related to better *financial behavior* in the older age group. Furthermore, the variables of retirement contribution were seemingly not viewed as important to the older group compared with their young and mature counterparts. Multidimensional interventions, especially targeting *behaviors* and *psychological* patterns, could, therefore, be recommended in advance to young and mature groups to prepare them to secure their old age and achieve FWB.

## 1. Introduction

Lifespans are far longer than they were a century ago. However, aging poses major challenges and will be a dominant theme for development in the 21st century. Today's young people will be part of the 2 billion older persons in 2050, and 80% of them will live in developing countries [1, 2]. Because of the increasing number of older people, inequality and economic insecurity among older adults are also major concerns [3]. Old-age pensions and access to healthcare are critical in reducing poverty and inequality among older people. However, nearly half (48%) of all people over pensionable age do not receive a pension. For many of those who do receive a pension, pension levels are inadequate. Thus, the majority of the world's older population has no income security [4].

Notably, Brazil is set to age rapidly over the next several decades, which will be driven by improved life expectancy and declining fertility rates [5]. The aging of the population will directly impact healthcare and social security, and the Brazilian public pension program (INSS) may be unable to provide sufficient income for the majority of people during retirement [6]. For instance, the Global Retirement Index [7] measures critical aspects of welfare in the retirement of older demographic retiree groups in countries across the world. In particular, Brazil comes in below average for the majority of factors, scores extremely low for “material well-being” (income equality index, income per capita index, and unemployment index), and ranks second to last in the Global Retirement Index, which is only ahead of India out of 44 countries analyzed. Moreover, in one of the economic indicators that best capture the multiple dimensions of economic and social progress created by the Organization for Economic Co-operation and Development, Brazil underperforms in average income, job, education, health, social connection, and life satisfaction [8].

This study evaluates the financial preparedness of various age groups for retirement in Brazil. The sample comprised 412 individuals aged 22–79 years. Data were derived from a survey to empirically validate the proposed relationships between *preparedness for retirement* and *resilience for the future* (financial well-being (FWB) outcomes) and among *demographic and socioeconomic* aspects, *behaviors and attitudes*, *knowledge and experience*, and “key” *psychological factors*. FWB is an essential component of aging well [9]; thus, its provision is a central requirement for overcoming the challenges of an aging population [10]. However, the literature provides neither an accepted definition of the FWB construct nor a standard measure of FWB [11, 12]. Therefore, a rigorously identified link among *knowledge* (financial literacy), *behaviors* (financial capability), *psychological factors*, and *demographic and socioeconomic factors* (e.g., age group) should be established in determining FWB. In this study, we mainly highlighted the importance of monitoring the *behavioral* and *psychological* aspects among age groups regarding saving for retirement as early as possible.

In a number of attempts to define FWB, several academic researchers on the topic note a two-component view of a current state and an expectation for the future. Notably, this expectation about financial security allows individuals to feel optimistic about handling future health-related events [12]. For instance, a study by the CFPB [11] posited a likely link between expected future financial security and overall well-being. The overall FWB used in the present study (which is a combination of meeting commitments, being financially comfortable, and *resilience for the future*) includes a separate outcome measure on how well people are financially prepared for retirement—*preparedness for retirement*—which has a direct connection with long-term thinking.

We adopted the *psychological resilience theory* in this study because it is a relatively positive and developmental outlook in that resilient people, according to the theory, tend to maintain high levels of well-being. Consequently, psychological resilience could be a personal factor for improving FWB and “preparedness for future retirement.” One commonly used measure of resilience is the self-report grit scale. *Grit* is a noncognitive trait, which is defined as perseverance and passion for long-term goals [13]. It represents a combination of educational, behavioral, and psychological factors and is dependent on the prospect of a better tomorrow than today, which is the connection made in this study. In addition, prior research reveals that *grit* scores vary with age; that is, adults with the most *grit* were in their sixties or older, and those with a bit of *grit* were in their twenties [14], as people may develop *grit* in the long run. This concept is the so-called maturity principle. However, Sigmundsson et al. [15] report relatively conflicting findings about grit increases-decreases across the life span; Sanders et al. [16] demonstrate significant evidence that the negative-parabolic shape of the grit-age profile is driven by generational variation, not age variation; and Morell et al. [17] claim that no evidence indicates that a consistent grit factor or factors exist across age groups. Thus, the need for a consensus about the maturity principle continues, and this issue remains open for further investigation.

Previous studies revealed a positive association between the total score for *grit* with various variables of successful aging (physical, emotional, social functioning, energy, and general health) and presented *grit* as a protective factor that promotes active adaptation to the developmental challenges of aging [18]. Notably, the results of Kim and Lee [19] demonstrated the larger influence of “perseverance” than those of health and economic level on successful aging. The authors argued that health and economic levels clearly exert a meaningful influence on the successful aging of the elderly, but the result of a subcomponent of *grit—*perseverance—as the most influential variable provides significant implications. Thus, scholars suggested the establishment of targets and psychological aspects for accomplishing these objectives as important for success in one's later years. Grit is also a useful construct to study because this trait affects long-term achievement that might be amenable to intervention. To aid in instilling and developing grit, the literature must clarify the natural motivators of gritty individuals [20]. Scholars also suggest prospective longitudinal studies across the life course to examine how individuals develop superordinate goals of such compelling personal significance that they inspire lifelong allegiance despite innumerable alternative pursuits and inevitable mistakes, failures, and other obstacles [21].

In summary, FWB is an emerging research area and remains scarce and scattered across disciplines [22], which are a trend that can also be observed in Brazil, which, thus, motivates further examination. This study tested the hypothesis that FWB is associated with *demographic and socioeconomic* aspects, *behavior and attitudes*, *knowledge and experience*, and *“key” psychological factors.* In particular, we are following two assumptions related to FWB research: H1: Socioeconomic aspects, financial knowledge, and psychological factors are explanatory variables for the financial behavior (financial capability) of different age groups. H2: Socioeconomic aspects and financial behavior are explanatory variables for the *preparedness for retirement*, *resilience for the future*, and FWB of different age groups.

## 2. Materials and Methods

### 2.1. Procedures

**Figure 1:**
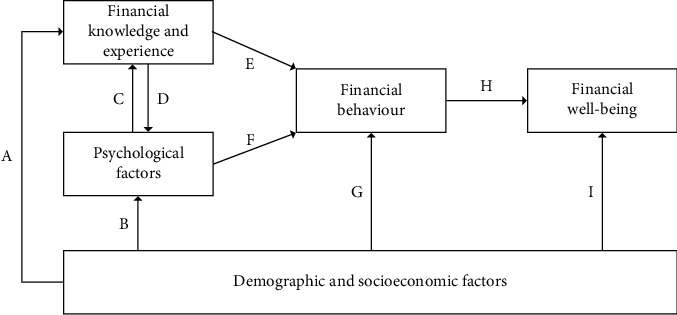
Financial capability and well-being model [24].

This study uses the methods of Kempson and Poppe [23] and Kempson and Evans [24]. Thus, the description of the method of the current study partially reproduces their wording. To evaluate the financial preparedness of different age groups for retirement in Brazil, we conducted ordinary least squares regression analysis using the financial capability and well-being model [23, 24], as shown in Figure 1. The independent variables of the model are *demographic and socioeconomic*, *behaviors and attitudes*, *knowledge and experience*, and *psychological factors*. The dependent variables are *preparedness for retirement* and *resilience for the future* as the outcomes of FWB. As an innovation to the methodology used in [23, 24], the current study also establishes *financial behavior* as an exploratory variable in the first moment of analysis. In addition, we propose *grit*, *resilience for the future*, and *retirement contribution* as variables relevant in explaining the *financial behavior* of the different age groups.

The *demographic and socioeconomic* data include age, gender, place of residence, employment status, schooling, income, and family type. The last group includes couples with children or dependents, living alone, living with parents or other relatives/friends, principal source of family income, own residence, solo parent, access to financial support from friends/family, and learned to manage/save money as a child. By controlling for age and other relevant variables, we can examine the effect of different *financial behaviors*, *knowledge*, and *psychological factors* in comparing people in similar socioeconomic situations.

Regarding *financial behavior*, people who save regularly are more likely to have an emergency fund, for example. Thus, better *financial behavior* results in a higher FWB. However, more is required to change behavior because a number of financial behaviors require prior knowledge. Thus, distinguishing between knowledge and behavior enables the identification of whether or not the issue is insufficient knowledge or whether barriers exist to the practical implementation of such knowledge. An important point is that financial knowledge is not the sole determinant of financial behavior because *knowledge and experience* are sometimes insufficient for changing behavior. Research suggests that psychological factors may be part of the “missing link” between knowledge and behavior (see, e.g., [25]).

The data for *psychological factors* are composed of the financial locus of control (the belief that one holds a level of control over one's financial situation); financial confidence; action orientation (tendency to procrastinate or not); attitudes toward saving, spending, and borrowing; long-term thinking; self-control; impulsivity control; and lack of concern about social status. The psychological factors are added in Part IV of the questionnaire (in the Supplementary Materials (available here)) and have been adopted from the studies by Kempson and Poppe [23] and Kempson and Evans [24].

*Psychological factors* influence a person's willingness to perform certain actions and thereby influence *knowledge and experience*. For example, a person with a low level of financial locus of control may believe that learning about money is useless. In turn, financial *knowledge and experience* can shift psychological factors; for example, acquiring financial *knowledge and experience* can increase financial confidence. In addition, *psychological factors* can affect financial behavior. For example, younger people tend to have shorter time horizons, but triggering a shift to longer-term thinking is possible by asking a person to imagine their life at retirement.

*Preparedness for retirement* is a separate outcome measure of the overall FWB. Its score alerts us to the public and private retirement savings of the population. Its results also illustrate the expectations and reliance on the governmental retirement income for workers in Brazil (INSS). Unfortunately, *preparedness for retirement* is a new measure, such that the availability of comparable data from other countries is limited. Nevertheless, it demonstrates the importance of examining this variable because it contributes to the available data and helps in future comparisons across countries. *Resilience for the future* is a subcomponent of FWB, which is heavily influenced by income and expenses. We measure it as the length of time in which individuals can cover their cost of living in the case that they lose their primary source of income without asking for a loan. *Preparedness for retirement* and *resilience for the future* are FWB outcomes [24].

We also included the personal factor of *grit* as an innovation in FWB research. We used the Brazilian-translated version of Duckworth's book [14] to measure the *grit* scale. In calculating the total grit score, all the points from the chosen options are added together, and the result is divided by 10. The maximum score on this scale is 5 (with a lot of grit), and the lowest score is 1 (no grit). The questions measuring grit elicit the consistency with which individuals dedicate themselves to long-term goals, which is the main reason for the relationship between the grit measure and this study. It is noteworthy that the Brazilian version of the grit scale has been previously used in the literature.

Finally, we collected data on current retirement contributions. Respondents could select more than one option from the following statements: I do not contribute to any regime; I contribute to general social security (INSS); I contribute to the social security for public servants; I contribute to a complementary plan (nonmandatory in Brazil); and I make investments to ensure my old age (e.g., real estate, fixed income, and government bonds). These items were constructed to reflect the Brazilian reality. Notably, INSS is mandatory for Brazilian workers with signed contracts, but the last two statements aim to collect data on the choice of making private investments to ensure financial security at retirement. In this manner, verifying whether or not age is a relevant factor to the attitude of making investments to secure one's old age is possible.

The Supplementary Materials contain the questionnaire. We used the online platform Google Forms to collect the data, which was divided into parts. The first part consisted of presenting the research and ensuring consent to participate. The second part aimed to collect information about the level of grit, followed by *resilience for the future*. We then collected data on *preparedness for retirement*, *psychological factors*, *financial knowledge and experience*, *behaviors and attitudes*, and *demographic and socioeconomic factors*.

Research projects involving humans in Brazil must comply with Resolution 466/12 of the Brazilian National Health Council. Therefore, we sought to ensure the rights of the participants and their free and informed consent to participate. The study conducted a pretest with two participants to collect their opinions about the wording and comprehensibility of the items, to identify possible inconsistencies, and to make adjustments accordingly. Subsequently, the questionnaire was disseminated via social media for one month (between April and May of 2022) and obtained 415 responses. After this period, we realized that the number of responses was already expressive and representative for the regions of Brazil, and we were no longer receiving responses.

Three participants failed to provide correct information on their age; therefore, the final sample included 412 valid responses. After the application of the questionnaire, a reliability test (Cronbach's alpha) was conducted for all original variables, presenting a result of 0.7614, which was above the lower limit of acceptability.

### 2.2. Data Analysis

To verify the relationships established in Figure 1, we used different quantitative models calculated from the responses to the questionnaires. First, we examined whether *demographic and socioeconomic*, financial *knowledge and experience*, and *psychological factors* influence *financial behavior*. Figure 1 suggests relationships among E, F, and G. Subsequently, we checked whether *financial behavior* and *demographic and socioeconomic* factors influenced *preparedness for retirement* and *resilience for the future*. The second step corresponds to rows H and I in Figure 1.

We use principal component analysis (PCA) to identify and construct a few of the variables of the survey, including the FWB measure. PCA is one of many analytical techniques used to explore patterns that naturally occur within the data. It considers the responses of participants to identify commonalities and reduce the variables of the underlying components. It is suitable for exploratory analysis without prior assumptions regarding the correlation of variables [26]. As criteria for selecting components, we used eigenvalues >1 [27].

We also conducted PCA using the 23 items in Part IV of the questionnaire (Supplementary Materials). First, we grouped the 23 questions in Part IV of the questionnaire into eight groups: locus of control, financial confidence, action orientation, saving/spending/borrowing attitudes, long-term thinking, self-control, impulsivity control, and lack of concern for social status. Then, we calculated a PCA from the eight groups created, which further reduced the items into two components, namely, PsyFactor1 and 2 (Table 1). The first pertains to a strong presence of factors related to one's control and confidence (called the psychological factor of control (PsyFactor1)). The second is more linked to the potential lack of concern for social status in conjunction with an orientation for action (called the psychological factor of action (PsyFactor2)).

## 3. Results

### 3.1. Descriptive Analysis

The *demographic and socioeconomic* variables, *behavior and attitudes*, *knowledge and experience*, and *psychological factors* were analyzed in view of the descriptive aspects (Figure 1). The youngest and oldest participants are aged 22 and 79 years, respectively, with an average age of 45 years. The majority are female (57.5%), have achieved college degrees of higher education (31.3%), and report a salary that exceeds the average salary of Brazilian workers (34.2%). Regarding the current employment status, the majority work in the public sector (34.9%). In addition, the majority of the participants live in their own homes (71.3%), are the primary source of family income (54.3%), and contribute to the social security of the Brazilian government (INSS; 50.2%). For a better understanding of the sample, Tables 2 and 3 provide a comprehensive image analysis.

Regarding *financial behaviors and attitudes*, an average participant reports taking an active role in making household financial decisions and planning to manage household finances (80.6%). The average participant also reports not borrowing for daily expenses (60.7%), uses credit carefully (67.9%), and is informed to make decisions and choose financial products (69.9%). On *financial knowledge and experience*, the average number of participants who reported knowing financial management (49.0%), comparing financial products (52.2%), having experience with financial management (38.9%), feeling included in financial matters (42.7%), and understanding the role of financial risk (58.2%) was lower but still represents the majority of the sample.

Furthermore, the study observed a strong relationship between the variables. The correlation between *financial behaviors and attitudes* and *financial knowledge and experience* is 0.50 for a two-tailed 5% critical value of 0.097. In addition, *behavior* and *knowledge* demonstrated a correlation with the *psychological factors of control* (0.48 and 0.51, respectively) and *action* (0.18 and 0.08, respectively) in this order.

**Figure 2:**
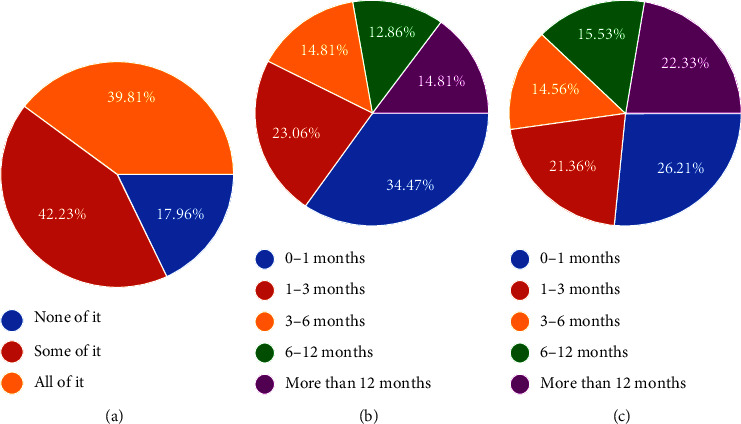
Resilience for the future.

*Resilience for the future* indicates that many people need to prepare for unexpected short-term expenses or decreases in income. The majority of respondents have less than a month's income savings, and 47% would only last three months without borrowing if their income decreased by one-third, as shown in Figure 2.

**Figure 3:**
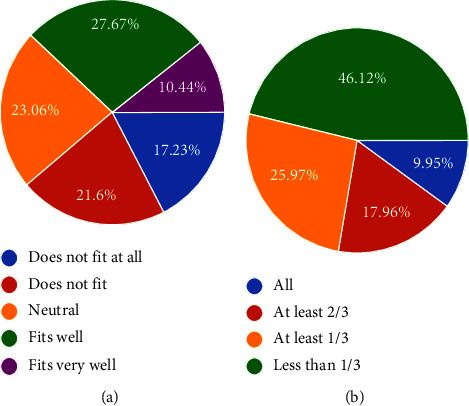
Preparedness for retirement.

**Figure 4:**
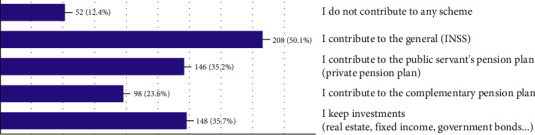
Retirement contribution.

Regarding *preparedness for retirement*, notably, while many believe they will have adequate retirement income without working, nearly 50% do not rely on state pension-INSS (Figure 3). Nonetheless, the results also illustrate low levels of private retirement savings across the Brazilian population. Even with low reliance on state pension, only 23.6% have a complementary pension plan and only 35.7% report private investments to secure their retirement (Figure 4).

### 3.2. Regression Analyses

Table 4 presents the regression analyses. Multiple regression obtained heteroskedasticity problems; thus, we used a model with correction (HC). In the first model, which includes the *demographic and socioeconomic*, financial *knowledge*, and *psychological factors* as explanatory variables for financial *behavior*, age was not associated with *behavior*. Better financial *behavior* was significantly associated with a high level of financial *knowledge*, PsyFactor1, PsyFactor2, being female, higher income, not living with others, and not being a single parent. Model 2 included *grit*, *resilience for the future*, and *retirement contribution* in the financial capability and well-being model. Interestingly, age displayed a negative relationship with *behavior*, and being the main source of family income appeared as an explanatory variable of better *financial behavior*.

Older age was related to *preparedness for retirement* (Model 3) and FWB (Model 5) but was not significant for *resilience for the future* (Model 4). Data not presented here revealed that age correlates with *resilience* at nearly zero (0.03), which indicates the lack of a statistical relationship despite being significant at 5% two-tailed for *preparedness* (0.36) and FWB (0.25). Out of the other socioeconomic variables, higher income, own residence, not being a single parent, contributing to a pension plan, and keeping private investments to secure old age were related to *preparedness for retirement*. Nevertheless, financial *behavior* was not an explanatory variable for *preparedness for retirement*. Better financial *behavior* was significantly associated with *resilience for the future* and FWB. Being male, higher income, living with others, not being a solo parent, being taught to handle money as a child, and retirement contribution variables were relevant for *resilience for the future* and higher income, not being a solo parent, and retirement contribution for FWB.

Tables 5–7 depict the results for the different age groups. The first age group consists of respondents aged less than 37 years. This age group corresponds to nearly one-third of the sample and mostly encompasses individuals who have recently entered the labor market. We expect people in this “young group” to retire in two or three decades, which mean that their time horizon until leaving the labor market is very long. The second group consists of people between the ages of 37 and 51 years. These respondents are likely to be in the labor market, and we refer to them as the “mature group.” Finally, people aged 52 year or older are expected to be close to retirement or already retired (the older group). In summary, financial *knowledge* and *PsyFactor1* were significantly related to better financial behavior for all age groups. The young and older age groups illustrated tendencies for being female to be related to better financial behavior and for being male to be related to *resilience for the future* and FWB. In addition, *grit* and *resilience for the future* appeared as explanatory variables for better financial *behavior* in the older group, and this model had the best explanatory power of all.

Notably, the attitude toward keeping private investments for retirement was an explanatory variable for better *preparedness for retirement*, *resilience for the future*, and FWB for the young and mature groups. However, it seems that the retirement contribution variables were not considered as important for the older group as for the mature and young groups. In summary, we noted that a number of results were more clear when the sample was divided according to age group. This finding indicates that the links between the proposed variables—grit, resilience, and retirement contribution—with the financial capability and well-being model proposed in Figure 1, which is the basis of this research, were better understood when viewed from the perspective of the different age groups.

**Figure 5:**
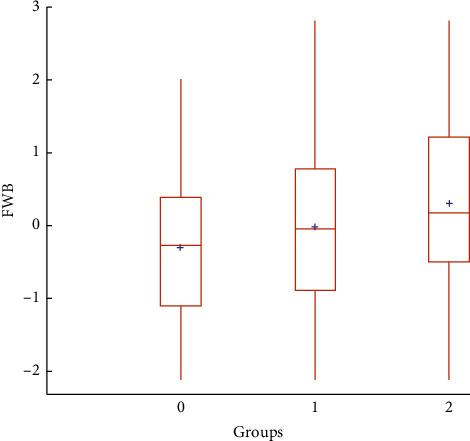
Financial well-being by age group. Source: prepared by the authors.

Figure 5 depicts the strong positive association between age and FWB. We can attribute this result to the strong association between age and *preparedness for retirement* because *resilience for the future* displayed no significant association with age (We found the absence of a relationship between age and resilience for the future in the research, which the researchers did not expect. The correlation between the two variables was 0.03. Furthermore, Model 5 indicated that the variable age was not significant. We further analyzed this model by adding age-interacting variables with significant independent variables. The result, not presented here, shows that of the eight independent variables alone, six lost significances, the exception being income, parents taught). On the one hand, these results are in agreement with a study conducted by the Consumer Financial Protection Bureau [28], among other studies of measures of FWB, in which older adults exhibited higher average scores for FWB than did younger adults. On the other hand, previous studies also showed an inverted U-shaped FWB across the life cycle (e.g., [29]). An unambiguous explanation for why FWB would be lower in old age is lacking, but vulnerability to emergency health expenditures and financial stress are two potential explanations. For example, Huang et al. [30] illustrated that subjectively and objectively measured financial stress is inversely associated with good self-reported health, quality of life, and life satisfaction and positively associated with self-reported depression among older adults in developing countries. Interestingly, previous studies also indicate that many old individuals are capable of maintaining psychological stability and well-being despite their experiences with poorer financial situations, among other adverse events [31]. High levels of resilience in older age are a phenomenon that is close to the concept that a few authors refer to as “the paradox of subjective well-being,” This concept states that levels of psychological well-being remain stable across old age (see, e.g., [32]). Hence, deepening one's knowledge about FWB, especially among older people, is seemingly of importance.

## 4. Discussion

This study aimed to evaluate the financial preparedness of different age groups for retirement in Brazil. The results demonstrated that *preparedness for retirement* was strongly related to older age. In addition, the *socioeconomic* aspects of higher income, own residence, not being a single parent, contributing to retirement plans, and keeping private investments to secure old age could explain a large proportion of the variance in *preparedness for retirement*. Nonetheless, better financial *behavior* was not an explanatory variable for *preparedness for retirement* in the overall empirical data. The analyses, including different age groups, additionally provided nuanced information on factors that influence the outcomes of FWB. For example, when comparing the regression models, better *financial behavior* was slightly related to *preparedness for retirement* in the young group. In the mature group, all variables of *contribution to retirement* were relevant for *preparedness for retirement*, whereas other socioeconomic aspects appeared as explanatory variables for *preparedness for retirement*, such as having children and being taught to manage money as a child, in the older group.

A number of classic behavioral explanations can be applied to the result of the relationship between age and *preparedness for retirement*. For example, Thaler [33] provided empirical evidence that people overvalue the more proximate satisfaction relative to the more distant ones. This behavioral bias can lead to a lack of concern about retirement because this future prospect for young people extends over a long period of time. Furthermore, one of the most consistent, prevalent, and robust biases documented in behavioral economics is “optimism bias.” In terms of predicting future events, people tend to overestimate the likelihood of positive events and underestimate the negative ones [34]. The phenomenon of the “illusion of control,” which predicts that factors from skill situations introduced into probability situations will cause people to feel inappropriately confident, is related to this notion [35]. These expectations of an inappropriately higher probability of personal success than those warranted by objective probability could also lead to a lack of concern about future retirement. Notably, Weinstein [36] suggested that these optimistic biases may be much less prevalent among older people. This explanation would be possible for the greater preparedness among older people.

The results between age and *preparedness* are in line with those of Munnell et al. [37], whose projections indicated that approximately 35% of Early Boomers (those born between 1948 and 1954) would lack an adequate retirement income, vis-a-vis 44% for Late Boomers (those born between 1955 and 1964), which increases to 48% for Generation Xers (those born between 1965 and 1974). However, the results of the present study are in contrast with those of a recent study by Qi et al. [38], which found no significant difference in retirement adequacy between Generation X (40–54 years old) relative to their Baby Boomer and Millennial peers. However, their results indicated that Generation X is better prepared for retirement than Millennials are in safer portfolio allocations. Notably, the Board of Governors of the Federal Reserve System of the United States [39] found that people have different savings thresholds to feel that they are on track to an adequate retirement. Furthermore, economic well-being substantially varies with the reason for retirement for those in retirement. This finding implies that a subjective as well as an objective evaluation of FWB is important when examining “successful” aging. In addition, goal clarity and involvement in financial activities were previously associated with the prediction of the retirement preparedness of working individuals [40]. This study additionally indicates that attitudes toward retirement contributions were associated with high levels of *preparedness for retirement*.

When comparing the financial *behavior* regression models, greater self-rated financial *knowledge* illustrated a strong relationship with better financial *behavior* for all age groups, which is in line with the results of previous studies [41, 42]. However, the findings of Pahlevan Sharif et al. [43] revealed that financial literacy did not influence the financial behavior of approximately 500 young adults in Malaysia. Furthermore, the inclusion of the variables, *grit* and *resilience for the future*, were relevant for better *behavior* in the older age group. These results follow the findings of Frankham et al. [44] in that skills related to personal agency, self-esteem, and coping were most frequently and reliably associated with the relationship between financial hardship and mental health outcomes. Research has increasingly viewed psychological resilience as a dynamic process by which people do not only bounce back from adversity and avoid the development of psychopathology but also ideally grow over time through experience [45]. To better understand this process and the conditions under which positive functioning and adaptive behaviors develop, the need emerges to understand the dynamic interplay and changes over the lifetime. In contrast with previous studies on financial behavior and age [46, 47], age was negatively associated with better financial *behavior* in the present study. However, the association between age and *behavior* did not remain when controlling for the different age groups, which highlights the importance of focusing on the association between age and behavior in future studies.

Out of the other *socioeconomic* variables included in the study, being female was highly associated with better financial *behavior*, while being male was significantly associated with *resilience for the future*, especially when controlling for age groups. Previous studies highlighted that the financial behaviors of men and women significantly differ [48]. In general, women were identified as less likely to engage in any of the financial behaviors than men do [49]. However, other studies reported no differences between men and women in this regard (e.g., [50]). In addition, although previous studies found that the teaching of parents directly informs the financial behavior of young adults [43], the current findings pointed to the importance of being taught to manage money as a child for *preparedness for retirement*, *resilience for the future*, and FWB among the older group. Nevertheless, apart from the retirement contribution variables, which were nonstatistically significant in improving the overall *behavior* (financial capability) model, and when controlling for the different age groups, a clear finding is that the attitude toward financially securing one's own old age should be considered to promote *preparedness for retirement*, *resilience for the future*, and FWB.

These findings support the notion that the cost-benefit of aging differs among age groups relative to certain *demographic and socioeconomic* aspects, *behavior and attitudes*, *knowledge and experience*, and *key psychological factors*. Notably, the socioeconomic aspects of being women, not having children, and being the main source of family income were associated with better financial *behavior* for the young group. Higher income, not being a single parent, and retirement contributions were related to better financial *behavior* in the mature group. Regarding the older group, we highlight the so-called maturity principle as a possible explanation for the strong positive association between the variables, *grit* and *resilience for the future*, with better financial *behavior*. Beyond that, financial *knowledge* and the *psychological factor of control* (PsyFactor1) were explanatory variables for better financial *behavior* of all age groups. Better financial *behavior* was not an explanatory variable for *preparedness for retirement* but for *resilience for the future* and FWB in nearly all age groups. Finally, the attitude of keeping private investments to secure old age was related to *preparedness for retirement*, *resilience for the future*, and FWB in the young and mature groups but not in the older group. We attribute this result to different “future time perspectives,” which would also be an interesting topic for future research.

### 4.1. Limitations

The study was based on a sample of individuals aged 22–79 years and contributed to an increased understanding of *preparedness for retirement* and *resilience for the future* of different age groups in Brazil. However, a few points need to be considered when interpreting the results. To a certain extent, the sample is wealthier than the overall Brazilian population, given that factors such as access to computers/smartphones and the Internet are likely to influence the probability of participating in the survey. For the same reason, we also expect the sample to report higher levels of education than those of the general Brazilian population.

Furthermore, the COVID-19 crisis, which began two years prior to the survey, may have depleted the savings of a number of people. Thus, low scores may be more likely for financial preparedness and resilience among those who experienced a decrease in income in the last two years, especially if they lost their jobs and are renting. For example, financial capability services in New Zealand have reported more clients experiencing more mental health issues and complex financial situations due to changing incomes due to the COVID-19 pandemic [35]. We collected data on the housing and employment status of the respondents; however, future research can verify the financial consequences of the COVID-19 pandemic in Brazil.

In addition, findings in the literature support that the transition from the third to the fourth age, which frequently occurs in individuals aged 80–85 years, entails extensive challenges to well-being [51]. Future research may further expand analyses by age. The findings of Näsman et al. [51] also highlighted that the subjective as well as objective evaluation of the economic situation is important when considering the life situation of older adults. Therefore, another interesting direction for future research would be the analysis of the subjective measure of FWB among different age groups in Brazil or in a comparison across countries. Furthermore, another interesting avenue for research would be a longitudinal study that will track whether or not and how FWB as well as SWB changes over the lifetime of an individual.

## 5. Conclusions

The results deepened the knowledge about the proposed links among *demographic and socioeconomic* aspects, financial *knowledge and experience*, *behaviors and attitudes*, and “key” *psychological factors* in determining an objective measure for FWB. The joint effect of self-control and confidence (*PsyFactor1*) as well as the lack of concern for social status in conjunction with an orientation for action (*PsyFactor2*) was suggested as “key” psychological factors that influence better financial *behavior*. In addition, *grit* and *resilience for the future* seemingly improved the financial *behavior*/capability model for the older group. We mainly highlight the importance of monitoring the *behavioral* and *psychological* aspects among different age groups to save for retirement as early as possible and avoid the so-called maturity principle, which can lead to low savings toward old age. In addition, by providing empirical data from Brazil, this research contributes to the international comparison in this field of study.

## Figures and Tables

**Table 1:** Principal components analysis for psychological factors.

Components	PsyFactor1	PsyFactor2
Locus of control	0.383	0.173
Financial confidence	**0.405**	0.074
Action orientation	0.269	**−0.526**
Saving/spending/borrowing	0.371	0.241
Long-term thinking	0.378	0.320
Self-control	**0.402**	−0.104
Impulsivity control	0.377	0.021
Lack of concern social status	0.182	**−0.719**

Source: prepared by the authors. The bold values indicate *p* < 0.05.

**Table 2:** Descriptive analysis (*n* = 412).

Alternatives	Frequency (%)	*N*
Gender		
Female	57.52	237
Male	42.48	175
Home location		
Center-west	61.89	255
Other	38.11	157
Employment status		
Retired	13.11	54
Unemployed and looking for work	2.67	11
Housewife	0.97	4
Student	1.94	8
Not working due to long-term illness, disability, or other reason	0.49	2
Partially retired	3.64	15
Civil servant	34.95	144
Self-employed or entrepreneur	20.87	86
Work with a registered job in the private sector or third sector	21.36	88
Schooling		
Incomplete elementary school	0.00	0
Complete elementary school	0.49	2
Incomplete high school	0.49	2
Complete high school	4.13	17
Incomplete higher education	7.28	30
Complete higher education	19.66	81
Postgraduation (lato sensu)	31.31	129
Incomplete master's degree	4.13	17
Complete master's degree	15.29	63
Incomplete doctorate	6.31	26
Complete doctorate	10.92	45
Income		
Far below average	5.34	22
Below average	4.85	20
Average	21.84	90
Above average	33.74	139
Way above average	34.22	141
Family type		
Couple with children or dependents	49.27	209
Lives alone	17.72	73
Lives with parents or other relatives/friends	32.52	134
You are the main source of your family income	54.3	224
You live in your own residence	71.36	294
You are a single parent	16.50	68
You have access to financial support from friends and family	38.11	157
Your parents talked about managing money or saving when you were a child	49.27	203
Retirement contribution		
I do not contribute to any scheme	11.89	49
I contribute to the general social security system (INSS)	50.24	207
I contribute to the public servant's pension plan (private system)	34.95	144
I contribute to a supplementary pension plan	23.54	97
I have investments to secure my old age	35.44	146

Source: prepared by the authors.

**Table 3:** Age.

Mean	Minimum	Maximum	Standard deviation	Coefficient of variation
45	22	79	13.12	0.29

Source: prepared by the authors.

**Table 4:** Regression analyses (*n* = 412).

Depend	Model 1	Model 2	Model 3	Model 4	Model 5
	Behavior	Behavior	Preparedness	Resilience	FWB
Model	HC	HC	HC	HC	HC
Const	2.603^*∗∗∗*^	2.664^*∗∗∗*^	−0.109	−1.041	−2.332^*∗∗∗*^
Behavior			0.049	0.774^*∗∗∗*^	0.162^*∗∗∗*^
Knowledge	0.225^*∗∗∗*^	0.233^*∗∗∗*^			
PsyFactor1	0.154^*∗∗∗*^	0.129^*∗∗∗*^			
PsyFactor2	0.086^*∗∗∗*^	0.088^*∗∗∗*^			
Grit		−0.017			
Resilience future		0.011			
Sex	−0.201^*∗∗∗*^	−0.227^*∗∗∗*^	0.235	0.613^*∗∗*^	0.133
Age	−0.004	−0.006^*∗∗*^	0.040^*∗∗∗*^	0.006	0.019^*∗∗∗*^
School	−0.004	−0.008	−0.049	0.070	−0.030
Income	0.073^*∗∗*^	0.050	0.180^*∗∗*^	0.591^*∗∗∗*^	0.233^*∗∗∗*^
With children	−0.122	−0.074	−0.014	−0.115	−0.063
Lives alone	−0.030	−0.013	−0.071	0.497	0.195
Lives others	−0.174^*∗∗*^	−0.172^*∗∗*^	−0.253	0.746^*∗∗*^	0.125
Main_source_	0.106	0.152^*∗∗*^	0.021	−0.462	−0.016
Own residence	−0.032	−0.017	0.325^*∗∗*^	−0.414	0.067
Single parent	−0.191^*∗∗*^	−0.202^*∗∗*^	−0.483^*∗∗*^	−0.952^*∗∗*^	−0.444^*∗∗∗*^
Fin support	−0.075	−0.041	0.009	−0.713^*∗∗*^	−0.170^*∗*^
Parents_taught	0.074	0.068	0.208	0.492^*∗*^	0.138
Any scheme		−0.133	−0.234	−1.071^*∗∗*^	−0.296^*∗*^
INSS		0.065	0.188	−0.114	0.040
Public servants		0.063	0.631^*∗∗∗*^	−0.278	0.125
Private prev		−0.052	−0.098	0.543	0.059
Private invest		0.092	0.373^*∗∗*^	1.773^*∗∗∗*^	0.568^*∗∗∗*^
R2	0.478	0.552	0.262	0.394	0.436
Fc	24.205^*∗∗∗*^	21.783^*∗∗∗*^	7.750^*∗∗∗*^	14.173^*∗∗∗*^	16.904^*∗∗∗*^

Source: prepared by the authors. *Note.* HC: heteroscedasticity correction. Model 1 describes the association between behavior, knowledge, PsyFactor1, PsyFactor2, and the socioeconomic variables. Model 2 includes additionally grit, resilience to the future, and retirement contribution as explanatory variables to behavior. Model 3 describes the association between preparedness, behavior, socioeconomic, and retirement contribution variables. Model 4 is similar to the previous one, but resilience is the explained variable, and in model 5 FWB. ^*∗∗∗*^*p* < 0.1, ^*∗∗*^*p* < 0.05, and ^*∗*^*p* < 0.01.

**Table 5:** Less than 37 years old (*n* = 138).

Depend	Model 6	Model 7	Model 8	Model 9	Model 10
	Behavior	Behavior	Preparedness	Resilience	FWB
Model	HC	HC	HC	HC	HC
Const	2.673^*∗∗∗*^	2.705^*∗∗∗*^	1.097	1.325	−2.491^*∗∗∗*^
Knowledge	0.155^*∗∗∗*^	0.169^*∗∗∗*^			
Behavior			0.297^*∗*^	0.615^*∗∗*^	0.224^*∗∗*^
PsyFactor1	0.186^*∗∗∗*^	0.185^*∗∗∗*^			
PsyFactor2	0.113^*∗∗*^	0.123^*∗∗*^			
Grit		−0.008			
Resilience future		0.011			
Sex	−0.272^*∗∗*^	−0.328^*∗∗∗*^	0.086	1.222^*∗∗∗*^	0.120
Age	0.014	0.005	0.012	−0.096	0.009
School	−0.039	−0.018	−0.075	−0.056	−0.053
Income	0.058	0.069	0.097	0.837^*∗∗∗*^	0.243^*∗∗∗*^
With children	−0.255^*∗∗*^	−0.277^*∗∗*^	−0.262	0.171	0.012
Lives alone	−0.198	−0.209	0.015	1.214^*∗*^	0.336^*∗*^
Lives others	−0.395^*∗∗∗*^	−0.374^*∗∗∗*^	−0.012	1.171^*∗*^	0.303^*∗*^
Main source income	0.154	0.196^*∗*^	−0.064	−0.620	0.149
Own residence	0.045	0.102	−0.007	−0.932^*∗∗*^	0.185
Single parent	−0.216	−0.083	0.532	−2.220^*∗∗∗*^	−0.560^*∗∗*^
Fin support	−0.211^*∗∗*^	−0.217^*∗∗*^	−0.037	−0.862^*∗∗*^	−0.052
Parents taught save	0.084	0.100	−0.076	−0.137	−0.143
Any scheme		−0.096	−0.704	−0.091	0.010
INSS		0.002	−0.412	0.981	0.094
Public servant scheme		−0.054	0.531	1.463	0.495^*∗*^
Private prev		−0.131	−0.028	0.430	−0.140
Private invest		−0.054	1.073^*∗∗∗*^	2.576^*∗∗∗*^	0.910^*∗∗∗*^
R2	0.650	0.680	0.403	0.559	0.617
Fc	15.085^*∗∗∗*^	11.120^*∗∗∗*^	4.471^*∗∗∗*^	8.370^*∗∗∗*^	10.639^*∗∗∗*^

Source: prepared by the authors. ^*∗∗∗*^*p* < 0.1, ^*∗∗*^*p* < 0.05, and ^*∗*^*p* < 0.01.

**Table 6:** Aged between 37 and 51 years (*n* = 133).

Depend	Model 11	Model 12	Model 13	Model 14	Model 15
	Behavior	Behavior	Preparedness	Resilience	FWB
Model	HC	HC	HC	HC	HC
Const	2.893^*∗∗∗*^	3.345^*∗∗∗*^	−1.902	3.472	−2.462^*∗∗∗*^
Knowledge	0.152^*∗∗*^	0.164^*∗∗*^			
Behavior			0.004	−0.153	−0.005
PsyFactor1	0.198^*∗∗∗*^	0.149^*∗∗∗*^			
PsyFactor2	0.027	0.069			
Grit		−0.062			
Resilience future		−0.016			
Sex	−0.193	−0.185	0.008	−0.766^*∗*^	−0.152
Age	−0.009	−0.012	0.062^*∗∗*^	−0.057	0.032^*∗*^
School	−0.023	−0.053	−0.025	0.150	−0.002
Income	0.142^*∗∗*^	0.137^*∗*^	0.180	0.666^*∗∗∗*^	0.151^*∗*^
With children	−0.126	−0.088	−0.131	−0.024	−0.075
Lives alone	0.236	0.167	0.111	0.000	−0.206
Lives others	0.099	−0.022	−0.197	0.628	−0.313^*∗*^
Main source	−0.061	−0.249^*∗*^	0.017	−0.429	−0.190
Own residence	−0.214	−0.209^*∗*^	0.800^*∗∗∗*^	−0.538	0.167
Single parent	−0.136	−0.256^*∗*^	−0.951^*∗∗*^	−1.508^*∗∗*^	−0.841^*∗∗∗*^
Fin support	−0.052	−0.071	0.175	−0.377	0.124
Parents taught	0.162	0.093	0.097	0.357	0.265^*∗*^
Any scheme		−0.062	−0.357	−0.215	−0.120
INSS		0.286^*∗*^	0.679^*∗*^	0.195	0.333
Public servant		0.441^*∗∗*^	1.315^*∗∗∗*^	0.430	0.780^*∗∗∗*^
Private prev		0.068	−0.631^*∗∗*^	0.883^*∗∗*^	−0.020
Private invest		0.226^*∗*^	0.662^*∗∗*^	2.295^*∗∗∗*^	0.456^*∗∗∗*^
R2	0.493	0.572	0.372	0.468	0.517
Fc	7.584^*∗∗∗*^	6.694^*∗∗∗*^	3.758^*∗∗∗*^	5.574^*∗∗∗*^	6.781^*∗∗∗*^

Source: prepared by the authors. ^*∗∗∗*^*p* < 0.1, ^*∗∗*^*p* < 0.05, and ^*∗*^*p* < 0.01.

**Table 7:** Older than 51 years old (*n* = 141).

Depend	Model 16	Model 17	Model 18	Model 19	Model 20
	Behavior	Behavior	Preparedness	Resilience	FWB
Model	HC	HC	HC	HC	HC
Const	1.502^*∗∗*^	1.342^*∗∗*^	−1.814	−6.855 ^*∗∗∗*^	−3.457^*∗∗∗*^
Knowledge	0.294^*∗∗∗*^	0.293^*∗∗∗*^			
Behavior			0.104	1.524^*∗∗∗*^	0.418^*∗∗∗*^
PsyFactor1	0.177^*∗∗∗*^	0.077^*∗∗∗*^			
PsyFactor2	0.117^*∗∗*^	0.159^*∗∗∗*^			
Grit		0.198^*∗∗*^			
Resilience future		0.064^*∗∗∗*^			
Sex	−0.349^*∗∗∗*^	−0.139	−0.084	1.286^*∗∗∗*^	0.330^*∗*^
Age	0.011	−0.003	0.025	0.065^*∗*^	0.017
School	0.021	0.025	0.062	0.165	−0.017
Income	0.041	0.026	0.573^*∗∗∗*^	0.594^*∗∗∗*^	0.349^*∗∗∗*^
With children	0.072	0.072	0.722^*∗∗*^	0.118	0.023
Lives alone	−0.076	−0.089	0.972^*∗*^	1.872^*∗∗∗*^	0.646^*∗∗*^
Lives others	−0.088	−0.093	−0.027	0.036	−0.139
Main source income	0.269^*∗∗*^	0.189^*∗*^	−0.167	−1.305^*∗∗∗*^	−0.234
Own residence	−0.160	−0.297^*∗*^	−0.105	0.710	0.227
Single parent	−0.256^*∗*^	−0.125	0.073	0.897	0.050
Fin support	0.017	0.089	−0.368	−0.795^*∗*^	−0.331^*∗*^
Parents taught save	−0.084	−0.051	0.871^*∗∗∗*^	1.002^*∗∗∗*^	0.353^*∗∗*^
Any scheme		0.105	−0.144	−3.667^*∗∗∗*^	−0.673^*∗∗*^
INSS		0.117	0.847^*∗∗*^	−1.036^*∗*^	0.040
Public servant scheme		0.069	0.308	−2.166^*∗∗∗*^	−0.183
Private prev		−0.086	−0.152	0.573	0.072
Private invest		0.024	−0.613^*∗*^	0.336	0.049
R2	0.612	0.800	0.388	0.629	0.455
Fc	13.153^*∗∗∗*^	21.406^*∗∗∗*^	4.299^*∗∗∗*^	11.510^*∗∗∗*^	5.651^*∗∗∗*^

Source: prepared by the authors. ^*∗∗∗*^*p* < 0.1, ^*∗∗*^*p* < 0.05, and ^*∗*^*p* < 0.01.

## Data Availability

The questionnaire data used to support the findings of this study have been deposited in the GitHub repository (https://github.com/EduardAugusta/data).

## Supplementary Materials

Supplementary Materials Appendix: online questionnaire.
