# Supplementary material for: The Cost-Benefit of Aging: Financial Capability and Well-Being across Age Groups in Brazil
Source: J Aging Res. 2023 Oct 10;2023:2020189. doi: 10.1155/2023/2020189 (PMC10581847; doi:10.1155/2023/2020189)
Supplement: Supplementary Materials — Appendix: online questionnaire. [file 2020189.f1.docx]

Dear participant,

You are being invited to participate in a survey for the final work of a doctoral thesis developed in the Graduate Program in Accounting at the University of Brasilia (PPGCont- UnB).

The approximate response time is 10 minutes. The data from this research are confidential and will be jointly analyzed without the possibility of identifying any of the participants.

In case of doubts or suggestions, please contact the researcher by the e-mail eduarda.augusta.sales@gmail.com.

We thank you in advance for your participation.

Eduarda Augusta

PPGCont/UnB

**Do you agree to participate in this survey?**

( ) Yes

( ) No

**Part I**

1. New ideas and projects sometimes distract me from previous ones.
2. Setbacks don’t discourage me. I don’t give up easily.
3. I often set a goal but later choose to pursue another one.
4. I am a hard worker.
5. I have a difficulty maintaining my focus on projects that take more than a few months to complete.
6. I finish whatever I begin.
7. My interests change from year to year.
8. I am diligent. I never give up.
9. I have been obsessed with a certain idea or project for a short time but later lost interest.
10. I have overcome setbacks to conquer an important challenge.

[*Grit: Five-point scale items (“nothing to do with me” to “totally me”). In analysis, statements 1, 3, 5, 7, and 9 are reversely coded].*

**Part II**

1. How much of an unexpected expense equivalent to one month’s income could you cover with money you have readily available?

2. What is your financial reserve volume in terms of number of months of income? (Short-term financial resources that you can readily count on)

3. How long could you cover a one-third drop in your income without having to borrow?

[*Resilience for the future: Question 1 - Three-point scale items (“nothing,” “something,”* and *“all”) Questions 2 and 3 - Five-point scale items (“0−1 month,” “1–3 months,” “3−6 months,” “6−12 months,”* and *“more than 12 months”)]*.

**Part III**

1. I will have adequate retirement income without working

2. Degree of reliance on INSS for retirement income

[*Preparedness for retirement*: *Question 1 - Five-point scale items (“it doesn’t really fit” to “fits very well”). Question 2 - Four-point scale items (“total,” “at least ⅔,” “at least ⅓,”* and *“less than ⅓”]*.

**Part IV**

Financial well-being is not completely determined by income. Behaviors, attitudes, and knowledge can change financial well-being outcomes. This section focuses on your self-perception; therefore, there are no right or wrong answers. WATCH OUT FOR CHANGES IN THE SCALES!

1. I can very well determine what will happen to me.

*[Five-point scale item (“it doesn’t really fit” to “fits very well”]*

2. My financial situation is largely out of my control.

*[Five-point scale item (“fits very well” to “it doesn’t really fit”)]*

3. When I make plans, I do everything I can to succeed.

*[Five-point scale item (“it doesn’t really fit” to “fits very well”]*

4. Level of confidence to manage money on a daily basis

*[Five-point scale item (“*very unconfident*” to “*very confident*”)]*

5. Level of confidence in planning for the financial future

*[Five-point scale item (“*very unconfident*” to “*very confident*”)]*

6. Level of confidence to decide on financial products and services

*[Five-point scale item (“*very unconfident*” to “*very confident*”)]*

7. When I have a difficult decision to make, I tend to put it off for another day.

*[Five-point scale item (“fits very well” to “it doesn’t really fit”)]*

8. When I have to do something important that I don’t like, I do it immediately.

*[Five-point scale item (“it doesn’t really fit” to “fits very well”)]*

9. When I have to choose between many options, I find it difficult to make up my mind.

*[Five-point scale item (“fits very well” to “it doesn’t really fit”)]*

10. I’d rather cut back on spending than use a credit card for what I can’t pay for every month.

*[Five-point scale item (“I strongly disagree” to “I strongly agree”)]*

11. I prefer to spend rather than save for contingencies

*[Five-point scale item (“I strongly agree” to “I strongly disagree”)]*

12. I find it more satisfying to spend than to save.

*[Five-point scale item (“fits very well” to “it doesn’t really fit”)]*

13. My focus is on the long term.

*[Five-point scale item (“it doesn’t really fit” to “fits very well”)]*

14. I live more for today than tomorrow.

*[Five-point scale item (“fits very well” to “it doesn’t really fit”)]*

15. The future will take care of itself.

*[Five-point scale item (“fits very well” to “it doesn’t really fit”)]*

16. I am good at resisting temptation.

*[Five-point scale item (“it doesn’t really fit” to “fits very well”)]*

17. I think it’s hard to break unwanted habits.

*[Five-point scale item (“fits very well” to “it doesn’t really fit”)]*

18. I do things without giving them much importance.

*[Five-point scale item (“fits very well” to “it doesn’t really fit”)]*

19. I am impulsive.

*[Five-point scale item (“fits very well” to “it doesn’t really fit”)]*

20. I say things without thinking about them.

*[Five-point scale item (“fits very well” to “it doesn’t really fit”)]*

21. I care about how other people see me.

*[Five-point scale item (“fits very well” to “it doesn’t really fit”)]*

22. I am concerned about social status among the people I meet.

*[Five-point scale item (“fits very well” to “it doesn’t really fit”)]*

23. I want other people to respect me.

*[Five-point scale item (“fits very well” to “it doesn’t really fit”)]*

**Part V**

Gender:

( ) Female

( ) Male

( ) I prefer not to say

2. Age

Answer: _____

3. State of residence:

( ) AC, AL, AP, AM, BA, CE, DF, ES, GO, MA, MT, MS, MG, PA, PB, PR, PE, PI, RJ, RN, RS, RO, RR, SC, SP, SE, TO, Abroad.

4. How would you describe your current professional status?

( ) Retired

( ) Unemployed and looking for work

( ) Homeowner

( ) Student

( ) No work due to long-term illness, disability, or other reason

( ) Partially Retired

( ) Public Servant

( ) Working as a freelancer or entrepreneur

( ) Work with a signed contract in the private sector or the third sector

5. Your education

( ) Incomplete Primary Education

( ) Complete Primary Education

( ) Incomplete High School

( ) Complete High School

( ) Incomplete College Education

( ) Complete College Education

( ) Post-graduation (lato sensu)

( ) Incomplete Master’s Degree

( ) Complete Master’s Degree

( ) Incomplete PhD

( ) Complete PhD

6. According to the latest Pnad Contínua (Continuous National Household Sample Survey), the average income of workers in Brazil was R$ 2,489. Considering this figure, would you say that your salary, compared with that of the average Brazilian, is,

( ) Far below average

( ) Below average

( ) Average

( ) Above average

( ) Way above average

7. Regarding your financial behaviors and attitudes

i. You play an active role in household financial decision making

ii. You take an active role in planning the management of your household finances

iii. You do not borrow for day-to-day expenses

iv. You use credit with caution

v. You are informed to make decisions and choose financial products

*[Five-point scale items (“it doesn’t really fit” to “fits very well”)].*

8. Regarding your financial knowledge and experience

i. You have knowledge about financial management

ii. You compare financial products

iii. You have experience with financial management

iv. You feel included in financial matters

v. You understand what financial risk is

*[Five-point scale items (“it doesn’t really fit” to “fits very well”)]*

9. Regarding your family type

i. Couple with children or dependents

ii. You live alone

iii. You live with parents or other relatives/friends

iv. You are the main source of your family income

v. You live in your own residence

vi. You are a single parent

vii. You have access to financial support from friends and family

viii. Your parents talked about managing money or saving when you were a child

*[Yes/No items]*

10. Regarding your retirement contribution. If necessary, choose more than one option.

I don’t contribute to any scheme

I contribute to the general social security system (INSS)

I contribute to the public servant’s pension plan (private system)

I contribute to a supplementary pension plan

I have investments (e.g., real estate, fixed income, government bonds) to secure my old age
